# Burden and Determinants of Anemia among Under-Five Children in Africa: Systematic Review and Meta-Analysis

**DOI:** 10.1155/2022/1382940

**Published:** 2022-09-11

**Authors:** Sisay Eshete Tadesse, Aregash Abebayehu Zerga, Tefera Chane Mekonnen, Abay Woday Tadesse, Fozia Mohammed Hussien, Yitbarek Wasihun Feleke, Melaku Yalew Anagaw, Fanos Yeshanew Ayele

**Affiliations:** ^1^Department of Nutrition and Dietetics, School of Public Health, College of Medicine and Health Sciences, Wollo University, Dessie, Ethiopia; ^2^Department of Public Health, College of Health Sciences, Samara University, Samara, Ethiopia; ^3^Department of Epidemiology and Biostatistics, School of Public Health, College of Medicine and Health Sciences, Injibara University, Injibara, Ethiopia

## Abstract

**Introduction:**

Globally, anemia among under-five children is a serious public health problem. Even if there are pocket studies here and there, there is limited evidence on the pooled prevalence of anemia among under-five children in Africa. Therefore, the aim of this study was to determine the pooled prevalence and determinants of anemia. *Methods and Analysis*. This systematic review and meta-analysis was done following the PRISMA guidelines. A comprehensive search was made in PubMed/MEDLINE, Cochrane Library, HINARI, and Ethiopian Journal of Health Development for studies published since 2009. It was supplemented with Google Scholar search. Study selection, data extraction, and quality of studies were assessed by eight reviewers. The Cochrane *Q* test and *I*^2^ test statistic were used to test the heterogeneity of studies. A random-effects model of DerSimonian-Laird method was used.

**Result:**

A total of 37 articles were included in this systematic review and meta-analysis. The pooled prevalence of anemia among under-five children in Africa was 59% (95% CI: 55, 63). Being female (AOR = 0.71; 95% CI: 0.57, 0.87), maternal education (AOR = 1.47; 95% CI: 1.31, 1.66), residence (AOR = 0.80; 95% CI: 0.67, 0.95), and family size (AOR = 0.93; 95% CI: 0.89, 0.98) were the determinants of anemia among African under-five children. *Conclusion and Recommendation*. This pooled study revealed that anemia was a severe public health problem. Sex, maternal education, residence, and family size were the determinants of anemia. Therefore, anemia prevention strategy should include sex consideration, educating mothers through youth education, area specific intervention, and encouraging birth spacing.

## 1. Background

Anemia among under-five children is defined as a hemoglobin level <11 mg/dl or children with hematocrit less than 33% [1]. Worldwide, anemia among under-five children is a major public health problem [2]. Globally, 20 million infants were born with low birth weight (LBW) every year. Nearly, 3.6 million of them died before celebrating their 28 days, of whom almost two-thirds were located in Sub-Saharan Africa and Southern Asia [3]. The effect of anemia can extend up to postpartum period and even newly delivered baby may suffer from a reduced iron store problem up to one year [4]. In developing countries, 46–66% of children under the age of five were affected by anemia [3]. African and Asian regions were the major contributor for a high burden of anemia [5].

The rapid growth and cognitive development of children make them more vulnerable for the development of anemia [6]. The consequences of iron deficiency anemia (IDA) during childhood include growth retardation, reduced school achievement, impaired motor and cognitive development, and increased morbidity and mortality. Mental impairments at early age are thought to be irreversible and the consequences may continue even after treatment, reinforcing the importance of early detection and prevention [7, 8].

The causes for anemia among under-five children are complex. Among these, low birth weight, undernutrition, poor socioeconomic status, household food insecurity, duration of breast feeding, poor dietary iron intake, poor maternal educational status, diarrhea, fever, poverty, poor sanitation and hygiene, monotonous diet, parent's level of education, and maternal anemia were the commonest contributors for under-five anemia [9–13].

Despite the numerous interventions done so far by the government of African countries and other concerning stakeholders, anemia among under-five children is still a severe public health concern [14–19]. Even though many independent pocket studies have been conducted in the region, the results were inconsistent and the prevalence varies significantly between studies [20–22]. In Africa, the pooled prevalence and determinants of anemia among under-five children have not been yet done. Assessing the pooled result will help to inspire the government's commitment and increase the social and resource mobilization in order to enhance the implementation of evidence based interventions for culminating the effect of anemia among under-five children in particular and the nation in general. Therefore, the aim of this study was to determine the pooled prevalence and determinants of anemia among under-five children in Africa. The findings of this study will help policy makers, program planners, health care providers, and concerned stakeholders to work more on anemia in order to reduce the prevalence of anemia, its consequences, and complication among under-five children.

Prompt identification and treatment of anemia lead to overall improvement of population health outcomes, improved physical exercise performance, and well-being that results in enhanced economic productivity.

## 2. Methods and Materials

### 2.1. Patient and Public Involvement

All under-five children in Africa were involved in this study.

### 2.2. Eligibility Criteria

All studies that reported prevalence and determinants of anemia among under-five children in Africa using English language and gray literatures were included. For estimating the prevalence of anemia, studies with cross-sectional design were included, while, for pooling the determinants of anemia, cross-sectional and case-control studies were included in the study. While studies whose full texts cannot be accessed after trying to contact the primary investigator within 3 months, descriptive studies, systematic reviews of the effects of an intervention, review articles, conference abstract and editorials were excluded from the study.

### 2.3. Search Strategies

This systematic review and meta-analysis was performed according to the Preferred Reporting Items for Systematic Review and Meta-Analysis (PRISMA) guidelines [23]. The study was conducted following the Joanna Briggs Institute (JBI) criteria. Data bases such as MEDLINE (via PubMed), EMBASE, and Cochrane Library, SCOPUS, HINARY, and Google Scholar were used to extensively search the relevant articles conducted since January 1, 2009. Gray literatures were also included by manual search.

### 2.4. Search Terms Used

The strategy applied to search articles from the electronic data bases was (anemia) OR (“iron deficiency anemia”) OR (“low hemoglobin level”) AND (determinants) OR (“associated factors”) (“Under Five Children”) AND (Ethiopia) OR (Eritrea) OR (Kenya) OR (Angola) OR (Benin) OR (Botswana) OR (“Burkina Faso”) OR (Burundi) OR (Cameroon) OR (“Cape Verde”) OR (“Central African Republic of Chad”) OR (Comoros) OR (Congo) OR (“Côte d'Ivoire”) OR (Djibouti) OR (“Equatorial Guinea”) OR (Gabon) OR (Gambia) OR (Ghana) OR (Guinea) OR (“Guinea-Bissau”) OR (Lesotho) OR (Liberia) OR (Madagascar) OR (Malawi) OR (MAli) OR (Mauritania) OR (Mauritius) OR (Mozambique) OR (Namibia) OR (Niger) OR (Nigeria) OR (Réunion) OR (Rwanda) OR (“Sao Tome and Principe”) OR (Senegal) OR (“Seychelles”) OR (Sierra Leone) OR (Somalia) OR (“South Africa”) OR (“Sudan”) OR (Swaziland) OR (Tanzania) OR (Togo) OR (Uganda) OR (“Western Sahara”) OR (Zambia) OR (Zimbabwe).

### 2.5. Data Extraction

After obtaining the full text of all articles, duplicates were screened and removed from the citation manger. Based on the eligibility criteria, eight reviewers (SE, AA, TC, AW, MY, FM, FY, and YW) independently reviewed the studies by title, abstract, and full article. Those included and undecided studies were further assessed by reading the full text. Studies that were not eligible based on the full text assessment were excluded and reasons were described for their exclusion in combination with the PRISMA flow diagram to summarize the selection procedure [23]. Studies that passed through this selection process were included in this study. Discrepancies between authors were resolved through discussion and consensus. The study characteristics (author, year of publication, region, target group, sample size, study design, response rate, and children with anemia), subject recruitment procedures, count data with (2 × 2 tables), crude odds ratio (where count data were not found), and population characteristics were extracted by using extraction sheet developed with Microsoft Excel 2013.

### 2.6. Quality Assessment and Risk of Bias

The Joanna Briggs Institute (JBI) critical appraisal check list was used to assess the quality of each paper. During data extraction, eight investigators independently performed the quality assessment. The quality scores of six data extractors were averaged. Any disagreement between investigators was solved by discussion and consensus. Finally, studies with higher scores (>50%) were included in the systematic review and meta-analysis.

### 2.7. Data Synthesis and Analysis

Data were analyzed using STATA version 14.0. The pooled proportion was calculated to estimate the prevalence of anemia. The pooled odds ratio (OR) with 95% CI was determined to estimate the determinants of anemia among under-five children. The degree of heterogeneity was checked by Cochran *Q* and *I*^2^ statistics. The Cochrane *Q* statistic was considered significant, if the *P*value is <0.10, while the *I*^2^ statistics at least 50% was considered to be significant [24, 25]. Since the variation between the study findings is significant, a random-effects model with 95% confidence interval was used. Heterogeneity was checked by running metaregression, subgroup analysis, and sensitivity analysis. Subgroup analysis was performed based on sex and study setting (region). Funnel plots analysis, Egger weighted regression, and Begg rank correlation tests were done to detect publication bias (*P* < 0.05 was considered as a suggestive of statistically significant publication bias) [25, 26].

### 2.8. Registration and Reporting

This systematic review and meta-analysis was registered in the PROSPERO with a CRD number of 42020150881.

### 2.9. Ethical Clearance

This study was reviewed and approved by institutional review board of College of Medicine and Health Sciences, Wollo University.

## 3. Result

**Figure 1:**
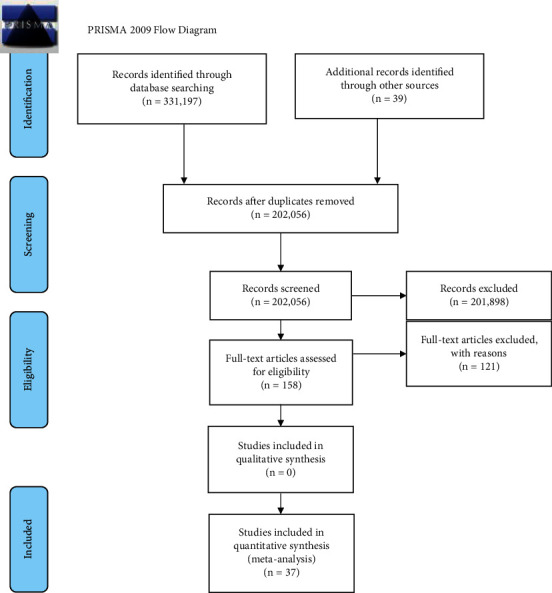
Preferred reporting items for systematic review and meta-analysis, June 2020.

A total of 331,236 articles were retrieved by literature search (Figure 1). Of these, 129,180 were excluded because of duplication, 201,898 did not have any relation with the aim of this study, and 121 did not meet the eligibility criteria. Finally, only 37 articles were included in this systematic review and meta-analysis. All included articles were full text and done using cross-sectional study design with one cohort [27] and three case controls [28–30]. The sample population varied from 210 [28] to 8,260 [31] children aging between 0 and 59 months. In this study, a total of 67,647 under-five children were included. The overall information regarding the prevalence of anemia was obtained from twenty African countries. These countries were Benin [32], Cameroon [33], Cape Verde [34], Congo [22], Ethiopia [14, 15, 31, 35–42], Gambia [43], Ghana [18, 28, 44], Guinea [20, 45], Kenya [19, 46], Lesotho [47], Malawi [48], Mozambique [29], Nigeria [49, 50], Rwanda [21], Senegal [17], Sierra Leone [51], South Africa [27], Tanzania [16, 30, 52–55], Togo [56], and Uganda [57–59] (Table 1).

### 3.1. Prevalence of Anemia among Under-Five Children in Africa

**Figure 2:**
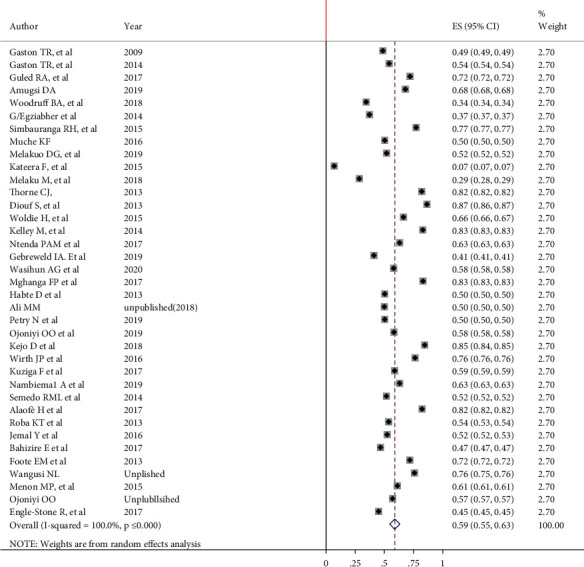
Forest plot for pooled prevalence of anemia among under-five children in Africa, 2009–2020.

The overall pooled prevalence of anemia among under-five children in Africa was 59% (95% CI: 55, 63). The true variability among studies other than chance was 100% (*I*^2^ = 100%, *P*value = 0.000). The lowest prevalence was observed in Rwanda 7% (95% CI: 7%, 7%), while the highest prevalence was observed in Senegal 87% (95% CI: 86%, 87%) (Figure 1). A study done in Rwanda did not include the milder form of anemia. This may be the reason for the lowest report of anemia prevalence in Rwanda (Figure 2).

To deal with the possible sources of heterogeneity, subgroup analysis was done by sex and region (study setting). The analysis result showed that heterogeneity still exists in both parameters mentioned above. In terms of region, the sources of heterogeneity were Ethiopia, Tanzania, Lesotho, Ghana, and Uganda.

**Figure 3:**
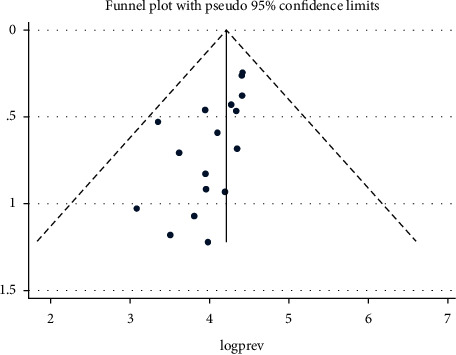
Funnel plot to detect the publication bias regarding prevalence of anemia, 2009–2020.

The following funnel plot appears asymmetric; even if it indicates the presence of publication bias, it was not statistically significant (Figure 3).

### 3.2. Sensitivity Analysis

#### 3.2.1. Determinants of Anemia among Under-Five Children in Africa

The result of this systematic review and meta-analysis indicated that sex of a child, maternal educational status, residence, and family size were the pooled determinants of anemia among under-five children in Africa. Being female is a protective against anemia among under-five children (AOR = 0.71; 95% CI: 0.57, 0.87), Mothers who were unable to read and write were 53% times more likely to have anemic child (AOR = 1.47; 95% CI: 1.31, 1.65). Those children from rural setting were 20% less likely to be affected by anemia as compared to children from urban setting (AOR = 0.80; 95% CI: 0.67, 0.95). Under-five children from a family size of less than five were 7% less likely to be affected by anemia (AOR = 0.93; 95% CI: 0.89, 0.98) (Table 2).

## 4. Discussion

This study was aimed at estimating the pooled prevalence and determinants of anemia among under-five children in Africa by reviewing the existing pocket studies. Based on the finding of this study, the pooled prevalence of anemia among under-five children in Africa was 59%. This finding is in line with a global prevalence of anemia [60]. According to the classification of World Health Organization (WHO), it was categorized under severe public health problem [61]. This finding suggests that, based on the current pace, it is difficult to achieve the global 50% reduction of anemia by 2025 in Africa [62].

This study showed that sex was a significant predictor of anemia among under-five children. Being female is protective against anemia among under-five children. The possible explanation for anemia discrepancy by sex could be due to the state of rapid growth of male children compared to females in the first months of life which increases their micronutrient requirement including iron, which cannot be met by diet alone [63]. If this physiological state is not compensated with iron rich complementary foods, risk of iron deficiency anemia will be higher in male children as compared to females.

This finding revealed that maternal education was a significant predictor of anemia among under-five children. Mothers with informal education were 53% more likely to have child with anemia. This finding is in line with a systematic review and meta-analysis study conducted in Ethiopia [64]. This might be because mothers with no formal education may not understand the introduction of scientifically sound feeding practices and are less likely to follow the recommended child feeding practices [65]. In addition, mothers with no education were negatively affecting the socioeconomic status of households which in turn limits food purchasing power and is a strong predictor for nutritional outcomes of children. Hence, their access to hem iron source food is limited [66]. In order to tackle the effect of anemia in children, nutrition education is suggested for mothers [67].

This study indicated that residence was a significant predictor of anemia among under-five children. Those children from rural setting were 20% less likely to be affected by anemia as compared to children from urban setting. This may be because mothers in the rural setting will breastfed their children exclusively till six months of age and continue breastfeeding till 24 months and more. Since iron in breast milk is more likely to be absorbed and utilized by the child's body, it will contribute for the normal stores of iron, which will help in reducing anemia among under-five children. In order to turn on the health loss due to anemia in children, UNICEF and WHO jointly recommended adequate breastfeeding practices [67]. But the finding of this study is inconsistent with a systematic review and meta-analysis study conducted in Ethiopia [64].

This study showed that family size was a significant predictor of anemia among under-five children in Africa. Those children from a household size of <5 were 7% less likely to be anemic as compared to their counterparts. This could be because large family size is associated with food insecurity. The lesser the families are, the more likely adequate and diversified diet can be afforded, which is rich in iron [68].

Some of the limitations of this study were articles published only in English language that were included. This may affect the prevalence estimation of anemia. Another limitation of this study was articles which were conducted among pediatrics that were not included. The data were obtained from twenty African countries. However, the analyzed pooled prevalence may not fully represent the prevalence of anemia in Africa because there is lack of evidences in some parts of the region.

To conclude, based on this systematic review and meta-analysis, anemia was a severe public health problem among under-five children in Africa. Sex, maternal education, residence, and family size were the determinants of anemia among under-five children. Therefore, adequate intervention should be designed by considering sex and residence difference, addressing maternal illiteracy through youth education and nutrition education, and promoting birth spacing.

## Figures and Tables

**Table 1:** Summary of extracted studies on anemia among under-five children in Africa, 2009–2020.

Author	Publication year	Study setting	Target group	Study design	Sample size	Prevalence	Quality
Gaston et al.	2009	Lesotho	Under five	Cross-sectional	1295	49	76.5
Gaston et al.	2014	Lesotho	Under five	Cross-sectional	1139	54	76.5
Guled and Mamat	2017	Ethiopia	6–59 months	Cross-sectional	397	72.0	67
Amugsi	2019	Ghana	6–59 months	Cross-sectional	2451	68.0	88
Woodruff et al.	2018	Guinea	Under five	Cross-sectional	5681	34	79
G/Egziabiher et al.	2014	Ethiopia	6–59 months	Cross-sectional	568	37	70.57
Simbauranga et al.	2015	Tanzania	Under five	Cross-sectional	448	77	58.9
Muchie	2016	Ethiopia	6–59 months	Cross-sectional	7636	50	61.8
Melako et al.	2019	Ethiopia	6–23 months	Cross-sectional	477	52.0	66.5
Kateera et al.	2015	Rwanda	6–59 months	Cross-sectional	1882	7.0	75.55
Melku et al.	2018	Ethiopia	6–59 months	Cross-sectional	707	29	80.3
Thorne	2013	Guinea	6–59 months	Cross-sectional	872	82.0	64.3
Diouf et al.	2013	Senegal	9–15 months	Cross-sectional	245	87	73.5
Woldie et al.	2015	Ethiopia	6–23 months	Cross-sectional	346	66	56.4
Van Buskirk et al.	2014	Ghana	0–36 months	Cross-sectional	861	83	61.5
Ntenda et al.	2017	Malawi	6–59 months	Cross-sectional	2597	63	67.8
Gebreweld et al.	2019	Ethiopia	6–59 months	Cross-sectional	404	41	65.9
Wasihun et al.	2020	Ethiopia	6–9 months	Cross-sectional	610	58	68.8
Mghanga et al.	2017	Tanzania	0–59 months	Cross-sectional	303	83	74
Habte et al.	2013	Ethiopia	6–59 months	Cross-sectional	8260	50	57.5
Ali	2018	Uganda	Under five	Cross-sectional	1808	50	66.5
Petry et al.	2019	Gambia	Under five	Cross-sectional	1354	50	78.4
Ojoniyi et al.	2019	Tanzania	Under five	Cross-sectional	7916	58	82.1
Kejo et al.	2018	Tanzania	6–59 months	Cross-sectional	436	85	67.5
Wirth et al.	2016	Sierra Leone	Under five	Cross-sectional	710	76	64.3
Kuziga et al.	2017	Uganda	6–59 months	Cross-sectional	376	59	52.9
Nambiema et al.	2019	Togo	6–59 months	Cross-sectional	2890	63	55.6
Semedo et al.	2014	Cape Verde	6–59 months	Cross-sectional	993	52	60.8
Alaofè et al.	2017	Benin	6–59 months	Cross-sectional	681	82	53.5
Roba et al.	2013	Ethiopia	6–23 months	Cross-sectional	216	54	72.1
Jemal et al.	2016	Ethiopia	6–59 months	Cross-sectional	399	52	66
Bahizire et al.	2017	Congo	6–59 months	Cross-sectional	838	47	71
Foote et al.	2013	Kenya	6–35 months	Cross-sectional	858	72	79
Wangusi et al.	2016	Kenya	6–23 months	Cross-sectional	227	76	68.1
Menon and Yoon	2015	Uganda	Under five	Cross-sectional	3878	61	80.1
Ojoniyi O	2017	Tanzania	Under five	Cross-sectional	6592	57	84
Engle-Stone et al.	2017	Cameroon	12–59 months	Cross-sectional	291	45	63.3
Total sample size					69,253	59.0	

**Table 2:** Pooled determinants of anemia among under-five children in Africa, 2009–2020.

Variables		Pooled AOR (95% CI)	Heterogeneity			*P*value
			*I* ^2^	*Q* statistic	*P*value
Malaria falciparum	Yes	1.31 (0.85, 1.78)	95.5%	15.74	0.000	0.22
	No	1				

Sex	Female	0.71 (0.57, 0.87)	94.1	240.3	0.000	0.001
	Male	1				

Stunting	Yes	1.16 (0.94, 1.43)	0.00	2.79	0.99	0.17
	No	1				

Maternal education	Informal	1.47 (1.31, 1.65)	0.00	5.69	0.89	0.00
	Elementary	1.05 (0.89, 1.25)	0.0	2.00	0.99	0.62
	High school	1				

Diarrhea	Yes	1.44 (0.86, 2.42)	93.9	245.06	0.00	0.17
	No	1				

ANC follow-up	Yes	1.68 (0.46, 6.23)	0.0	0.08	0.43	0.43
	No	1				

Residence	Rural	0.80 (0.67, 0.95)	0.0	5.14	0.64	0.000
	Urban	1				

IFA intake	Yes	0.99 (0.63, 1.57)	0.0	0.00	0.73	0.44
	No	1				

Family size	<5	0.93 (0.89, 0.98)	0.0	4.19	0.52	0.004
	≥5	1				

Occupation	Unemployed	1.14 (0.86, 1.51)	0.0	1.77	0.98	0.38
	Employed	1				

## Data Availability

All the required data is included within the article.
